# A mobile health-assisted cardiovascular follow-up after pregnancy complications (MumCare): protocol for a randomized controlled trial

**DOI:** 10.3389/fgwh.2026.1889953

**Published:** 2026-10-13

**Authors:** Anne Cathrine Staff, Meryam Sugulle, Josef Noll, Miriam K. C. Sollie-Hoel, Belen Oteiza Aguirre, Bendik S. Fiskå, Gunvor Aasbø, Atle Klovning, Mette-Elise Estensen, Åsa Henning Waldum

**Affiliations:** 1 Division of Obstetrics and Gynaecology, Oslo University Hospital, Oslo, Norway; 2 Institute of Clinical Medicine, Faculty of Medicine, University of Oslo, Oslo, Norway; 3 Department of Technology Systems, University of Oslo, Kjeller, Norway; 4 Department of Public Health Science and Interdisciplinary Health Science, Faculty of Medicine, University of Oslo, Oslo, Norway; 5 ProCardio Centre of Innovation, Department of Cardiology, Rikshospitalet, Oslo University Hospital, Oslo, Norway; 6 Department of Cardiology, Oslo University Hospital, Oslo, Norway

**Keywords:** cardiovascular disease, diabetes mellitus, empowerment, gestational hypertension, preeclampsia, pregnancy, selfcare, telemedicine

## Abstract

**Objective:**

A history of hypertensive disorders of pregnancy and/or gestational diabetes is associated with increased risk of long-term maternal cardiovascular (CV) disease. Despite recommendations for preventive strategies, few women receive CV follow-up postpartum. We have developed a personalized digital mobile Health companion, the MumCare (Mum’s cardiovascular health for life) application, aiming at improving control of modifiable risk factors for CV disease and to bridge hospital and community health services. This paper describes the protocol for a MumCare randomized controlled trial in Norway.

**Methods:**

Study participation is offered to women at Oslo University Hospital with a diagnosis of hypertensive disorders of pregnancy and/or gestational diabetes. Following informed consent (either during pregnancy or within 4 weeks postpartum), a block randomization process assigns participants to one of two groups (access to the Mum Care app or not). The app provides educational material, allows women to track modifiable risk factors, and prompts feedback to registered blood pressures. It also prompts the women to book the nationally recommended follow-up at their general practitioner’s at 3–4 and 12 months postpartum. The primary study objective is to assess the effect of MumCare application access on 1-year follow-up rate with a general practitioner. Secondary objectives are to assess group differences at 14 months postpartum regarding: 1) satisfaction with the postpartum mHealth-assisted technology, 2) self-management evaluation, general self-efficacy; and at 14–18 months: 3) modifiable risk factors for CV disease (including hypertension, dyslipidemia, blood sugar control, smoking, weight and physical activity), CV non-invasive hemodynamics and CV-related biomarkers.

**Discussion:**

This randomized controlled trial tests the effect of a mobile Health-assisted follow-up of modifiable risk factors after pregnancy complications associated with premature CV disease in women. A study limitation is a risk of underrepresentation of recently immigrated women due to the restricted language options offered in the app.

**Study protocols registration:**

NCT05835596.

## Introduction

Worldwide, cardiovascular disease (CVD), in particular myocardial infarction and stroke, is a main cause of premature death for women and a major cause of disability (1). As recently summarized by the Lancet women and cardiovascular disease Commission, “women with cardiovascular disease remain understudied, under-recognised, underdiagnosed, and undertreated” (1). Prevention of CVD is a global priority, as incidence and health-related costs are increasing (2). Primary prevention of CVD includes prevention and reduction of risk factors, a strategy likely to be most efficient when started as early as possible (3).

Pregnancy can be seen as a clinically underexploited cardiovascular (CV) stress test for women (4). Epidemiological studies show an increased risk of maternal CVD following several pregnancy complications, including hypertensive disorders of pregnancy (HDP) (e.g., preeclampsia and gestational hypertension) and gestational diabetes mellitus (GDM) (4, 5).

The risk of experiencing a CV event (e.g., stroke, myocardial infarction) later in life is increased 2- to 8-fold for women with prior HDP, depending on the severity of the pregnancy complications (4–6). Two proposed theories for these associations, not excluding each other, include common risk factors and pregnancy-mediated pathways to CVD in women (5). We have previously shown that an increased risk for hypertension within 10 years after preeclampsia persists after adjustment for major CV risk factors (7), supporting our theory that HDP complications may accelerate adverse maternal CV changes (4, 5, 8, 9). A recent population-based study from Finland concluded that having experienced HDP was more important for the future risk of CVD, than a high polygenic risk score for several CV-related outcomes (10). This study does not rule out common genetic pathways to HDP and CVD but highlights a hypertensive pregnancy outcome as an important red flag for follow-up strategies and potential cardiovascular preventive intervention.

GDM is a common complication of pregnancy worldwide, with a rising incidence, affecting one in six births (11). Short-term consequences of GDM include increased risk for developing preeclampsia, as well as premature birth (12). Women with GDM have a 7-fold increased risk for progression to type 2 diabetes mellitus within 10 years after delivery (13, 14). As diabetes is a well-known risk factor for CVD, it is not surprising that GDM is also associated with increased risk for maternal CVD later in life (15).

Current CVD risk calculators are not useful for young women at increased risk after pregnancy complications (16), as these risk calculators neglect the impact of such pregnancy complications and often are not applicable to a population below 40 years of age. In older women, tests provide more adequate CVD risk scoring (17), but at this age preventive effects are likely less effective (3). Population-based studies from Denmark (18) and Norway (7) have shown that one third of women develop hypertension within 10 years following HDP. Middle age may therefore be too late to start intensified follow-up and efficient prevention of CVD risk. The risk of hypertension is high in the first postpartum years after HDP (19, 20) and women with GDM may also have identifiable CVD risk factors (21). Pregnancy represents a life-changing event where women may be particularly interested in their own and their offspring’s health. Pregnancy and the postpartum setting can therefore represent an optimal time point to empower women to improve their own CV health for life, as is the focus of the MumCare (Mum’s cardiovascular health for life) study.

In response to the American Heart Association’s 2011 seminal publication to adverse pregnancy outcomes being viewed as an independent risk factor for maternal CVD risk (22), the CoLAB (Global Pregnancy Collaboration) group developed in 2016 a pragmatic suggestion for clinical follow-up after such hypertensive pregnancies (5). This suggestion was simplified in the 2020 Norwegian national guidelines for obstetrics, published by The Norwegian Medical Association (23), and detailed in a patient information brochure (24). Current international guidelines for pregnancy complications like hypertensive disorders of pregnancy and GDM rarely detail suggestions for early preventive CVD strategies. A selection of these guidelines is shown in Supplementary Table 1. Updated guidelines from the European Society of Cardiology suggest “periodic screening for hypertension and DM (diabetes mellitus) should be considered” following preeclampsia and GDM (25). Obstetric-related guidelines [such as (26–28)] mostly lack specific recommendations for CVD follow-up after pregnancies complicated by hypertension. GDM guidelines generally suggest postpartum testing for persistent hyperglycemia and diabetes development [e.g., Hemoglobin A1c (HbA1c), fasting glucose or oral glucose tolerance test] (29, 30), however few guidelines explicitly suggest other postpartum follow-up procedures to reduce CVD risk. The chapter on management of GDM in the Norwegian obstetric guidelines (31) and corresponding patient information (32) recommend a standard first-year postpartum follow-up similarly to the suggested follow-up after HDP, followed by long-term personalized follow-up according to identified risk factors (23, 24).

World Health Organization (WHO) promotes digital health investments, emphasizing that “digital health investments should be coordinated to support continuity of care”. Electronic health (eHealth) is defined by the WHO as the use of information and communication technology for health (33). Mobile health (mHealth) is a component of eHealth, using mobile communication technology. Use of proactive reminder systems and mHealth technology warrants further research (34).

Telemonitoring and digital interventions have a potential to support self-management (35, 36). e- and mHealth assistance for health improvement is to our knowledge rarely offered postpartum. Our recent scoping reviews identified a lack of CVD preventive mobile application (app) interventions addressing the postpartum period following GDM (37) or HDP (38). Our hypothesis is that an eHealth-assisted approach could be beneficial for this group of women at increased risk for CVD. This group of young (fertile) women has in general a high IT (information technology) literacy, which would aid such a CV health strategy from the postpartum period. Some eHealth interventions have been designed to improve female health prior to pregnancy (39, 40). During pregnancy, e- and mHealth assistance have been less utilized, except for home monitoring of blood pressure (41–43). Some positively evaluated eHealth programs (44) have provided health care services during pregnancies at risk for hypertension (41–43) or GDM (45). Other recent eHealth studies have included, in addition to telemonitoring of blood pressure during pregnancies at risk, monitoring of other maternal clinical symptoms and signs as well as fetal telemonitoring (46–48). Home blood pressure monitoring is likely a useful screening modality in the detection and management of postpartum hypertension (49, 50).

A recent study from Norway has established that women with preeclampsia or GDM are motivated for CV follow-up after pregnancy (51), but a specific eHealth approach has not been explored in such a setting nationally or globally. Women in Norway expressed in interviews after such pregnancy complications that they wanted healthcarers to motivate them to lifestyle changes, and their partners expressed a wish to also participate in such a postpartum follow-up (51, 52). Our recent web survey from Norway indicated however that only 36% of women who have experienced HDP or GDM receive some preventive CV follow-up after such pregnancies (53). Other countries, like the USA, report even lower follow-up rates (54), supporting the need for a better transition between levels of healthcare.

In the MumCare study, we are conducting a randomized controlled trial (RCT) to test whether our recently developed eHealth MumCare app (55) promotes CV health optimization in premenopausal women after such pregnancy complications. The long-term goal is to prevent or postpone clinical signs of maternal CVD, by educating and empowering women and enabling modifiable risk factors to be discovered earlier through use of the MumCare app. Guideline-based preventive therapies and strategies would be offered when indicated, at an earlier time point in a woman’s life than offered today (e.g., by treating hypertension and hyperlipidemia).

The primary objective of the MumCare RCT is to assess whether the rate of the recommended 1-year postpartum follow-up at the general practitioner’s is increased with MumCare app access, compared to no app access. Secondary objectives are to assess satisfaction with this eHealth-assisted follow-up, as well as effects on self-management, general self-efficacy and modifiable risk factors for CVD (including hypertension, dyslipidemia, blood sugar control, smoking, weight and physical activity).

Our hypothesis is that systematic preventive measures starting postpartum through introduction of eHealth-supported programs can improve empowerment and maternal CV health trajectories. More specifically, our hypotheses are that the new mHealth-assisted program (the MumCare app*)* will be used by women offered this opportunity, and that these women will report increased self-management, general self-efficacy and physical activity, and that they will more often seek follow-up at the general practitioners (GP) at 3–4 and 12 months postpartum (as recommended in the national guidelines). We also hypothesize that modifiable risk factors for CVD and biomarkers related to increased risk for CVD will be more favorably modified after 14–18 months intervention time in app users, as compared to women not randomized to MumCare app access.

## Material and methods

### Setting

In Norway, antenatal, delivery and postpartum care are publicly funded and provided free of charge. The MumCare study is performed in south-eastern Norway, at Oslo University Hospital, which has the largest Obstetric Department in Norway, with 8,700 deliveries annually. The Department serves both primary hospital functions for the capital’s inhabitants but also serves as a referral center for regional (e.g., very preterm deliveries) and some national patient groups (e.g., pregnant women with congenital heart diseases).

In Norway, all residents are assigned a GP through the national *Regular General Practitioner Scheme (Fastlegeordningen)*. GPs provide comprehensive, long-term, and person-centered healthcare throughout the patient’s lifespan, ensuring continuity of care. Most Norwegian GPs are board-certified specialists in general practice or are in formal training for specialization. The Norwegian primary healthcare system is organized with the GP as the first point of contact for medical issues. GPs function as gatekeepers to specialist healthcare, coordinating diagnostic and therapeutic referrals and ensuring appropriate use of secondary care services. This role also facilitates continuity, equity, and cost-effective care across the healthcare system.

In the Norwegian national recommendations for follow-up after pregnancies associated with an increased epidemiological risk of CVD, like HDP or GDM, GPs play a key role. This role includes preventive counselling, risk assessment, and the early detection of preclinical CVD manifestations, including hypertension, hyperlipidemia, hyperglycemia, and overweight or obesity. When indicated, GPs refer patients to secondary care for further evaluation or treatment.

### User involvement

The MumCare study has involved user groups since the planning of the study, including local hospital advisory user group and user representatives from two relevant groups [The Norwegian SIDS (sudden infant death syndrome) and Stillbirth Society, LUB, and Adults with congenital heart disease, VMH]. We have previously described the co-creation process in developing the MumCare app (55), which included users and stakeholders in several stages prior to the final version of the MumCare app. In brief, our co-creation process, including qualitative interviews, showed that the relevant users welcomed mHealth-assisted follow up of personal CV risk factors (55). They also suggested several changes to functions and visual elements of the app, both prior to and during the app programming (55). These user group representatives have contributed with important user perspectives, confirming the need for the study and advising on recruitment and follow-up procedures.

The MumCare study and app has also obtained support, as described previously (55), from three Norwegian healthcare provider associations relevant to the MumCare study; The Norwegian College of General Practice, The Norwegian Society of Gynecology and Obstetrics, and The Norwegian Society of Cardiology.

### The MumCare mHealth app

MumCare is a novel postpartum mHealth app created for women with a recent history of HDP and GDM. It is based on the Norwegian obstetric guidelines (23, 31), and provides information in line with general health guidelines from the Norwegian Directorate of Health (56). The MumCare study is designed for testing the app the first 14–18 months postpartum, but the app has no technical limitation on the postpartum timeline use.

The MumCare app has three main parts; (1) an educational part [short videos on blood pressure (measurement instructions and clinical implication), health information and links to public available web sites], detailed in our previously published paper on the MumCare co-creation process (Table 1) (55); (2) the patient’s own health data (entered by the user herself) that represent modifiable risk factors for CVD (e.g., blood pressure, fasting/non-fasting blood glucose, weight, physical activity); and (3) a push reminder system.

**Table 1:** Inclusion and exclusion criteria for participation in the MumCare randomized controlled trial study.

	Inclusion criteria (all 1–4 required)	Definition
1	Age ≥18 years old	
2	Ongoing or recent pregnancy	• Pregnant or no more than 4 weeks postpartum
3	Ongoing or recent pregnancy complication of: Hypertensive disorders of pregnancy (HDP), and/or gestational diabetes mellitus (GDM)	• HDP: either ○ Gestational hypertension: New-onset hypertension (at least 140 mmHg systolic and/or at least 90 mmHg diastolic) from ≥20 gestational weeks during the present pregnancy (26). ○ Preeclampsia: Gestational hypertension in addition to new-onset proteinuria and/or at least one other maternal sign of organ dysfunction, according to 2018 International Society for the study of Hypertension in Pregnancy criteria (74). • GDM: as defined by Norwegian national guidelines (Fasting plasma glucose: ≥ 5.3-6.9 mmol/L, or 2 h glucose value (oral glucose tolerance test following 75 gram oral glucose): ≥ 9.0-11.0 mmol/L (32, 75)
4	Possessing a smartphone and able to understand app use	

	Exclusion criteria	
1	Preexisting chronic/essential hypertension, diabetes mellitus	Chronic/essential hypertension, prepregnancy diabetes mellitus and/or other chronic diseases requiring cardiovascular postpartum follow-up by specialist or general practitioner
2	Language barrier to the MumCare inclusion process and study app information	Patient not understanding Norwegian or English

GDM, gestational diabetes mellitus; HDP, hypertensive disorders of pregnancy.

The educational part of the app presents information about these pregnancy complications and their association to premature CVD, as well as preventive recommendations. It provides links to patient brochures (24, 32) that summarize the current recommendations for preventive measures and follow-up of the Norwegian obstetric guidelines for HDP (23) and GDM (31). The app incorporates The Norwegian Directorate of Health’s general recommendations for primary CVD prevention, including healthy nutrition and physical activity (56), as well as the CV benefits of breastfeeding (57).

The data collection part of the app invites women to track their own blood pressure (BP) measurements using a BP device [validated for home use (58) and available to her without costs at study entry]. Any BPs, physical activity, blood sugar values (fasting and non-fasting) and weights registered by the women are tracked on longitudinal graphs. The app generates personalized feedback on the blood pressures that the patients register, according to her previous track record of registered blood pressures, as described by us previously (55).

The push reminder system of the app works on two levels, as described in our co-creation paper (55). Firstly, it provides prompts for when to measure a new blood pressure (according to previously registered blood pressures). Secondly, it prompts the woman to book a basic CV follow-up visit at her GP at 3–4 and 12 months postpartum (based on the woman’s entry of her date of delivery into the app), in line with Norwegian obstetric guidelines (24, 32). She is also prompted to register own modifiable CV risk factors at the same time points.

The MumCare app is programmed in almost identical app versions in Norwegian and English, the latter differing only in presenting links to US and UK web sites, in settings where no English text version was available on the relevant Norwegian web sites. The educational videos made for MumCare (on the measurement and clinical significance of blood pressure) were also made available in English. Both the Norwegian and English video versions have available subtitles (in line with universal design principles).

### Selection/treatment of subjects

Inclusion and exclusion criteria are shown in Table 1 which also displays the clinical definitions used for the target patient groups (HDP and GDM).

### Intervention arms

#### Interventional model description

Study consenting women (ongoing pregnancy or maximum 4 weeks postpartum of a pregnancy complicated by one or both of the inclusion diagnoses) are randomized prospectively. Women are randomized to MumCare app access or not. There is no masking of the randomization, as the woman herself is aware of her app access or not.

#### MumCare app access in the active RCT arm

Access to the MumCare app from study recruitment represents the active intervention arm of the MumCare RCT. The group randomized to have access to the MumCare app will, in addition to standard care, have access to the app for at least up to 18 months postpartum, until the scheduled follow-up at the hospital. They are also provided with a blood pressure device validated for home monitoring (59).

#### The control arm of the RCT

Women randomized to not have access to the MumCare app will receive standard care, which includes being provided oral and written information according to delivery ward routines after the woman’s pregnancy complication.

### Recruitment

MumCare study information with QR (Quick response) codes are available on posters across the localization of the Obstetric Department at Oslo University Hospital, as well as on hospital and national clinical study web sites (60). Eligible women are either contacted by the study personnel at the Oslo University Hospital or the women contact the study personnel themselves for inclusion. The study personnel will have sufficient time to answer questions and explain further the consequences for the particular woman. The study recruiter will also assess if the woman has a smartphone and adequate technical understanding for MumCare app use. Oral and written information is provided. All eligible women are offered participation. After informed written consent, case report form completion and blood sampling, the patients are randomized by a block randomization process assigning participants to one of two groups: either access to the MumCare app or no access to the app. In each block of four, two of the codes (ending with an a) will enable the women to use the MumCare app downloaded on her phone from App Store (for Apple units) or Google Play (for Android units). The two remaining codes (ending with b) will give the women access to information about the MumCare study (that she participates in) but does not give her access to the MumCare app features. The combination of the blocks of access codes (aabb, bbaa, abab, baba, baab, abba) and their ranking during the recruitment of the planned 400 participants are computer generated.

### Technical solutions for the MumCare app

The MumCare app provides women with a secure and personalized support for monitoring and controlling their personal CV risk factors and health following pregnancy.

The digital infrastructure, including the app and a supportive cloud infrastructure, is based on in-depth security and consent-driven privacy assessment. Core to the security- and privacy-design principles are encryption and de-identification of data. University of Oslo’s Department of Technology System supervised the security design and assessment. The woman with MumCare app access generates a unique PIN (personal identification number) code (not known to the study personnel), that opens the app for registrations and access to information.

User-generated app data will normally (after the RCT study) only be stored in the app itself. For the purpose of the RCT MumCare study, user-generated app data and questionnaire responses will be stored in the app as well as in secure servers at the University of Oslo (TSD; Service for Sensitive Data), in agreement with the user’s consent. The clinical data from hospital assessments will be stored at similar secure servers at Oslo University Hospital. Both servers and IT systems are in line with the requirements in Norwegian and European laws and regulations. The case report form, patient reported outcome measures (PROMs) and questionnaires are linked by a unique MumCare study code (5 digits).

dHealth, a Norwegian company specialized in health app development, programmed the MumCare app, ensuring secure IT solutions and data transfer to the University of Oslo servers.

### Outcome measures

All women in the MumCare study (i.e., both arms of the RCT) will be invited via SMS (short message service) to a web-based questionnaire 14 months postpartum. This is followed by a phone call the following business day with an invitation to a CV health evaluation at Oslo University Hospital at 14–18 months postpartum.

Table 2 provides an overview of the primary and secondary outcomes of the MumCare study.

**Table 2:** MumCare randomized controlled trial study outcomes (assessed 14–18 months postpartum): overview of primary and secondary outcomes.

Variable category	Variable	Definition/description	Assessed how	To be included in the statistical analyses of the primary outcome
Primary outcome	GP attendance 1 year postpartum	Has the participant attended or booked Cardiovascular risk screening at GP (as per guideline recommendation)	Telephone interview and digital questionnaires at 14 months postpartum	Yes
Secondary outcomes	Patient Reported Outcome Measures (questionnaires)	Questionnaires are listed in Table 3 and Table 4	Digital questionnaires at 14 months postpartum	No (descriptive analysis)
	Qualitative interviews of users and their GP (interviews)	Compare user experiences (health perception, empowerment, quality of life) with and without app use (for both the woman and her GP)	Assessed by MumCare study personnel during qualitative interviews 14–18 months postpartum	No (descriptive analysis)
	Modifiable risk factors for CVD 14–18 months postpartum	Listed in Table 5	Assessed by MumCare study personnel at clinical follow-up 14–18 months postpartum	No (descriptive analysis)
	CV hemodynamics 14–18 months postpartum	Listed in Table 5	USCOM: assessed by MumCare study personnel at clinical follow-up 14–18 months postpartum	No (descriptive analysis)
	Classical and novel CV-related biomarkers 14–18 months postpartum	Listed in Table 5	Assessed by MumCare study personnel at clinical follow-up 14–18 months postpartum	No (descriptive analysis)
	Descriptives of the app user group	App usage (frequency of use as well as use of information pages and responses to feedback on BP measurements) • The rate (and speed) of normalization of BP postpartum the first 6–12 months (or *de novo* hypertension) • Numbers and frequencies of BP entries postpartum, according to time since delivery and by blood pressure groups • How many warnings of elevated BP (and were new BP entered)	MumCare study personnel summarizing the automatically transferred user entered data from app users into secure data system at UiO	No (descriptive analysis)

BP, blood pressure; CV, cardiovascular; CVD, CV disease; EPJ, electronic patient journal; GDM, gestational diabetes mellitus; GP, general practitioner; UiO, University of Oslo; USCOM, ultrasonic cardiac output monitor.

The primary MumCare study outcome is defined as the rate of patients having attended the recommended postpartum CV follow-up at her GP around 12 months postpartum, as recommended by the national Norwegian (and international) guidelines after HDP (24) and GDM (32). This primary outcome will be assessed through the questionnaires sent by SMS as well as by telephone interviews at 14 months postpartum. Any GP visits within the first year postpartum for CV follow-up will be recorded, including visits at the recommended 3–4 months postpartum after HDP (24) or GDM (32). Non-responders will be reminded by phone, and a new SMS will be sent in case of still no response. At the end of the questionnaires, participants are informed of available help resources if they are in need for further mental support (e.g., from their primary health care provider or a Norwegian public mental health organization, the latter available 24/7).

Secondary MumCare study outcomes are listed in Tables 3–5. Table 3 lists the validated questionnaires (and their scoring systems and interpretations) used to assess PROMs in the electronic questionnaires (sent at 14 months postpartum). The validated questionnaires include the Health Education Impact Questionnaire (heiQ) (61), General Self-Efficacy (GSE) scale (62), and International physical activity questionnaire (IPAQ) (63). The group randomized to MumCare app access will also receive the System usability scale (SUS) questionnaire (64–66).

**Table 3:** MumCare study outcomes (assessed 14 months postpartum): validated patient reported outcome measures (PROMs) questions (and their scoring systems) collected digitally from study participants.

	Questionnaire (and references)	Description	Number of items	Scales
1	Health Education Impact Questionnaire (heiQ) (61)	Patient reported health-related self-management: health education impact questionnaire.	40 items representing 8 domains that evaluate immediate effects of self-management interventions.	1 (strongly disagree) to 4 (strongly agree). A higher score indicates better status, except for “emotional distress”, where higher score indicates higher distress.
2	General self-efficacy (GSE) scale (76)	Patient reported health-related quality of life. The GSE Scale measures optimistic self-beliefs in coping with the challenges of life: “refers to optimistic self-beliefs of being able to perform and control behaviors, and is linked with various physical and mental health outcomes”.	10 items.	4-point scale ranging from 1 (not at all true) to 4 (exactly true). Total scale ranges from 10 to 40. A higher score indicates more optimistic self-belief.
3	International physical activity questionnaire (IPAQ) (63)	Patient-reported last 7 days recall of physical activity, validated for use in the adult population.	4–7 questions (3 questions follow a positive answer to the previous question)	a) *Continuous scale*: The total physical activity metabolic equivalent (MET)-minutes per week is calculated. b) *Categorical scale*: The physical activity level is classified as “low”, “moderate” or “high”.
4	System usability scale (SUS) (66) -only presented to the group randomized to MumCare app access	The SUS (one commonly used questionnaire assessing eHealth applications) is a brief survey that provides subjective data regarding the perceived usability and overall acceptance of a product or service (here: the MumCare app). SUS is validated in English. The Norwegian version is based on a validated version in Danish (which closely resembles the Norwegian language).	10 questions	1 (strongly disagree) to 5 (strongly agree)

**Table 4:** MumCare study-specific questions (assessed 14 months postpartum): Non-validated questions (and their scoring systems).

	Question	Theme	Scale
Non-validated study-specific questions (7) to the group randomized to app use, with response alternatives and scales.			
1	Did you have experience using health apps before this pregnancy? (e.g., for lifestyle, blood pressure, weight, physical activity)	MumCare mobile application use	“Yes”, “no”, “don’t know”. If “yes”: an option to write a comment is given. (e.g., app name).
2	Was it useful that you could enter your own health data into the app?		Scale 1-5: 1 (not useful) 5 (very useful)
3	I would recommend the MumCare app to women after pregnancy complications (e.g., high blood pressure or gestational diabetes) for follow-up of their cardiovascular health		Scale 1-5: 1 (strongly disagree) 5 (strongly agree)
4	Did you have a cardiovascular health check-up with your general practitioner 3-4 months after giving birth? (e.g., for blood pressure, blood sugar, weight)	Postpartum follow up at the general practitioner’s	Yes”, “no”, “don’t know/don’t remember”. If “yes”: the respondent is asked if blood pressure and HbA1c were measured.
5	Did you have a cardiovascular health check-up with your general practitioner 1 year after giving birth? (e.g., for blood pressure, blood sugar, weight)		Yes”, “no”, “don’t know/don’t remember”. If “yes”; the respondent is asked if blood pressure, blood lipids and HbA1c were measured, if lifestyle recommendations were given. An option to write a comment is given.
6	Did you have cardiovascular health check-ups after giving birth, other than those already mentioned above?		Yes”, “no”, “don’t know/don’t remember”. If “yes”; the respondent is asked how many times, and is given the option to write a comment.
7	Do you currently take medication for the treatment of diabetes, high blood pressure or cardiovascular diseases?		Yes”, “no”, “don’t know”. If “yes”; the respondent is asked which ones and is given the option to write a comment.
Non-validated study-specific questions (8) to the group randomized to no app use, with response alternatives and scales.			
1	Did you have experience using health apps before this pregnancy? (e.g., for lifestyle, blood pressure, weight, physical activity)	Mobile application use in general	“Yes”, “no”, “don’t know”. If “yes”: an option to write a comment is given (e.g., app name).
2	Did you use health apps in this pregnancy? (e.g., for lifestyle, blood pressure, weight, physical activity)		“Yes”, “no”, “don’t know”. If “yes”: an option to write a comment is given (e.g., app name).
3	Would you have taken a more active role in your own health management after giving birth, if you had access to a health app designed for your pregnancy complication (e.g., high blood pressure or gestational diabetes)?		“Yes”, “no”, “don’t know”.
4	If a health app adapted to your pregnancy complication (e.g., high blood pressure or gestational diabetes) was available to you, would you have used it?		“Yes”, “no”, “don’t know”.
5	Did you have a cardiovascular health check-up with your general practitioner 3-4 months after giving birth? (e.g., for blood pressure, blood sugar, weight)	Postpartum follow up at the general practitioner’s	Yes”, “no”, “don’t know/don’t remember”. If “yes”; the respondent is asked if blood pressure and HbA1c were measured.
6	Did you have a cardiovascular health check-up with your general practitioner 1 year after giving birth? (e.g., for blood pressure, blood sugar, weight)		Yes”, “no”, “don’t know/don’t remember. If “yes”; the respondent is asked if blood pressure, blood lipids and HbA1c were measured, if lifestyle recommendations were given. An option to write a comment is given.
7	Did you have cardiovascular health check-ups after giving birth, other than those already mentioned above?		Yes”, “no”, “don’t know/don’t remember”. If “yes”: the respondent is asked how many times, and is given the option to write a comment.
8	Do you currently take medication for the treatment of diabetes, high blood pressure or cardiovascular diseases?		Yes”, “no”, “don’t know”. If “yes”: the respondent is asked which ones and is given the option to write a comment.

**Table 5:** MumCare clinical and biomarker variables assessed at follow-up 14–18 months postpartum in both study groups (women with or without MumCare app access postpartum). Data will be presented as mean/median values and/or rates of abnormal values by study group. Blood samples are drawn non-fasting, also for lipid evaluations (77, 78).

Clinical/biomarker variable	Definitions/values
Blood pressure (BP)	Normotension (or not); defined by the: a) AHA/ACC 2025 guidelines (79): a. Normal BP: < 120 mm Hg systolic and < 80 mm Hg diastolic; b. Elevated BP: 120-129 mm Hg systolic and < 80 mm Hg diastolic; b) ESC 2024-25 guidelines (80): a. Non-elevated BP: < 120 mm Hg systolic and < 70 mmHg diastolic; b. Elevated BP: 120-139 mm Hg or 70-89 mm Hg diastolic; c) Classical definition (81) of not having hypertension: a. < 140 mm Hg systolic and < 90 mm Hg diastolic.
Body mass index (BMI)	Normal weight (or not); defined as BMI (weight in kilos divided by height in square meters) 18.5-24.9 kg/m^2^.
Ultrasound Cardiac Output Monitor (USCOM)	USCOM device (ultrasonic cardiac output monitor; continuous-wave Doppler analysis of transaortic blood flow, measuring hemodynamics non-invasively) (82). Group differences in outcome variables will be described: Systemic Vascular Resistance, Cardiac Output, Stroke Volume, Heart Rate.
Blood sugar (glucose) (non-fasting, serum)	Elevated (or normal) non-fasting plasma blood sugar; defined as > 7.8 mmol/L (140 mg/dL, which is considered elevated and may indicate an increased CV risk (83). Any plasma blood sugar ≥ 11.1 mmol/L (≥ 200 mg/dL) is considered as a diagnostic criterion for diabetes mellitus [especially if accompanied by symptoms of hyperglycemia (84)]. Any of these values need further investigation and follow-up.
HbA1c (EDTA blood)	Normal values of hemoglobin A1c (HbA1c) (or not); defined as < 42 mmol/mol. Diabetes mellitus is diagnosed if HbA1c is ≥ 48 mmol/mol (6.5%) (85, 86) and will be referred for further care. HbA1c of 42–47 mmol/mol (5.7%–6.4%) indicates high risk for developing diabetes (and life-style intervention combined with annual HbA1c assessment is therefore recommended) (87).
HDL cholesterol (serum)	Normal (or not) level of serum HDL (high-density lipoprotein); defined as HDL ≥ 1.3 mmol/L or above (88). HDL < 1.3 mmol/L indicates high risk for developing cardiovascular disease. (89) HDL < 1 mmol/L is outside normal range (90).
Total cholesterol (serum)	Normal (or not) level of total serum cholesterol; defined as serum cholesterol ≤ 5 mmol/L. Total cholesterol > 5 mmol/L indicates increased risk for developing cardiovascular disease, assess treatment according to overall risk. Total cholesterol ≥ 7 mmol/L often indicates need of therapy (90).
LDL cholesterol (serum)	Normal (or not) level of LDL (low density lipoprotein) cholesterol; defined as LDL cholesterol < 3 mmol/L (91). LDL cholesterol > 3 mmol/L indicates increased risk for developing cardiovascular disease. LDL cholesterol > 4.8 mmol/L often indicates need of therapy (90).
Non-HDL cholesterol (serum)	Normal (or not) non-HDL cholesterol (calculated as total cholesterol minus HDL-cholesterol); defined in this context as 3.4 mmol/L. These recommended values are used for secondary treatment targets: < 2.2 mmol/L in patients with very high CV risk, < 2.6 mmol/L in patients with high CV risk and <3.4 mmol/L in patients with moderate CV risk (91).
Lp(a) (serum)	Normal (or not) Lp(a) (lipoprotein a); defined as < 75 nmol/L (< 30 mg/dL). Elevated values reflect mainly genetic risk for CVD (92, 93). A Lp(a) value > 75 nmol/L (> 30 mg/dL) is reported as elevated, but a Lp(a) value > 125 nmol/L (> 50 mg/dL) is considered a threshold for increased cardiovascular risk (94, 95).
TG (serum)	Normal (or not) level of serum TG (triglycerides); defined as non-fasting TG < 2.0 mmol/L. Values < 1.7 mmol/L indicate lower cardiovascular risk. (91) If > 4 mmol/L the test is recommended to be repeated in fasting state (96).
ApoB (serum)	Normal (or not) serum ApoB (apolipoprotein B); defined as ApoB > 1.4 g/L. The normal reference range for women is 0.5–1.4 g/L. High levels indicate increased risk for developing cardiovascular disease.
ApoA1 (serum)	Normal (or not) serum ApoA1 (apolipoprotein A1); defined as ApoA1 within the normal reference range for women: 1.1–2.0 g/L.
ApoB/ApoA1 ratio	Normal (or not) serum ApoB/ApoA1 ratio; defined as ApoB/ApoA1 ratio ≤ 0.6. In women, a ratio > 0.6 indicates increased risk of cardiovascular disease (88).
Waist-hip-ratio	Normal (or not) waist to hip ratio (both measured in same unit; in nearest full cm); defined as a ratio < 0.85 in women.
Waist circumference	Normal (or not) waist circumference (measured in nearest full cm); defined by: a) ACC/AHA and ESC as < 88 cm in women (97, 98). b) World Health Organization as < 80 cm (99).
Serum cardiovascular-associated biomarkers	Group differences in biomarkers will be described for: GDF-15 (Growth differentiation factor 15; elevated during preeclampsia) (100), hsCRP (high-sensitive C-reactive protein; a general inflammation marker that is an independent risk factor for CVD, and elevated in preeclampsia) (101), sHLA-G (soluble Human Leukocyte Antigen G; an immune modulating protein, low levels in preeclampsia, but increased level postpartum) (102), NT-proBNP [pro-B-type Natriuretic Peptide; well-established CV biomarker that may help identifying preclinical disease (103)], and other potential cardiovascular-related biomarkers (including PZP; pregnancy zone protein and SAA1; serum amyloid A1) (104)).

Table 4 lists the study-specific (non-validated) questions for the participants randomized to app access and those for the participants randomized to not having app access. Table 5 lists the clinical assessment outcomes, biomarkers and modifiable clinical risk factors for CVD assessed during the clinical follow-up 14–18 months postpartum at Oslo University Hospital. Additional blood samples (after the routine biochemistry analyses) are stored for future research studies related to maternal health after pregnancy, in agreement with patient consent.

Table 6 provides a timeline overview of patient recruitment, data collection, intervention and follow-up assessments of the MumCare study.

**Table 6:** MumCare RCT overview (SPIRIT table): overview of patient recruitment, data collection, intervention and follow-up assessments.

	Enrolment	Allocation			
Intervention TIMEPOINT	Day 1	Day 1	12 months pp	13–14 months pp	14–18 months pp
ENROLMENT:					
Eligibility screen (inclusion and exclusion criteria assessed)	X				
Informed consent	X				
Blood sampling for biobank	X				
Allocation (randomized to app access or not)		X			
INTERVENTIONS (app access):					
Randomized to MumCare app access		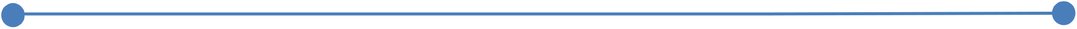			
Randomized to NO MumCare app access		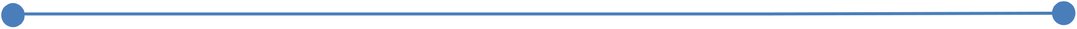			
ASSESSMENTS:					
Baseline variables	X				
Modifiable CV risk factors postpartum		For app users only: measure and register health -related data in the app (blood pressure, weight, physical activity, blood sugar)			
Primary outcomes: attended GP follow-up as per guidelines or not			X (assessed by phone call 13–14 months pp)	X	X
Secondary outcomes: PROMs				X	X
Secondary outcomes: Modifiable CV risk factors					X
CV hemodynamics					X
Blood sampling for biobank	X				X
Patient and GP perceived usefulness of the MumCare app (qualitative interviews)					X

CV, cardiovascular; GP, general practitioner; pp, postpartum; PROMs, patient reported outcome measures.

#### Qualitative studies

Some women (from both the HDP and GDM groups) will be invited to semi-structured individual interviews performed 14–18 months postpartum. These interviews will assess whether the access to the MumCare app (compared to no access) has affected the women’s postpartum period, self-management evaluation, general self-efficacy, and physical activity. Some random participating women randomized to app access are invited to waive their doctors’ obligation of confidentiality. The GPs caring for these women are invited through qualitative interviews to assess how they perceive that the MumCare app has affected their patient’s cardiovascular health self-management. The results of these qualitative studies with semi-structured interview plans will allow further refining of the next app version.

### Sample size

The main outcome (proportion of women visiting their GP in Norway at 12 months postpartum for CV follow-up) was unknown at study start. We recently performed a web-based survey in Norway (53), where we identified a self-reported follow-up of 36% after GDM or HDP (without a significant difference between the two self-identified groups). This study population was however biased, as the study invitation was sent by social media and was available (through QR codes) on printed posters at some of the largest hospital in Norway. If we nonetheless assume a follow-up of 35% vs. 50% for the two groups (participants without or with access to the MumCare health app), we would need to include 338 participants (169 in each of the two groups) (Sample Size Calculator), a power of 80% and a significance level of 5%, when the randomization is 1:1 (for app or non-app access). Due to an unknown rate of dropouts regarding patient reports on whether a GP visit at around one year postpartum has been undertaken or not, we decided to recruit 400 participants in the RCT MumCare study (either diagnosed with GDM and/or HDP). Recruitment was planned over 24 months (June 2024-June 2026), considering available clinical recruiters and eligible patient groups at the three delivery sites and two outpatient locations across the recruiting hospital (Oslo University Hospital).

For the qualitative interview studies (of participating women and their GPs), a purposeful sample of participants will be included, depending on the information power (67) of the interviews (likely 5–10 per the proposed interview groups).

## Data analysis

### Data handling, analysis and result reporting

After study inclusion, baseline data such as patient demographics, medical and obstetric history, and pregnancy anamnesis are collected and registered in a case report form before entering and coding the data in an electronic system. After delivery, remaining relevant data for the assessment of pregnancy outcome are registered from the hospital’s electronic patient journal system. Primary and secondary outcome data are registered at the 14 (phone interview and electronic questionnaire) and 14–18 months (clinical assessment) follow-up.

A unique MumCare study code links the information between the de-identified databases at the end of the RCT, to conclude whether MumCare app usage (objectively measured on the University of Oslo server) correlates with improved CV health and/or PROMs as well as the primary outcome (CVD preventive follow-up at the GPs one year postpartum).

The primary research question will be analyzed according to the intention to treat principle, to investigate the effect of *assigned* access to the MumCare app compared to no access. In this analysis, we will include all randomized study participants in the groups they were assigned to, regardless of adherence or withdrawal. Missing data will be described, but no imputation will be performed in the main analysis. In addition, we will perform per-protocol analyses to investigate the effect of *using* the app, including only participants who adhered to the assigned treatment arm (app users) compared to those assigned to the non app access group. We here defined “adherence” as registration in the MumCare app of any health data beyond the first 4 weeks after app access (randomization). We will analyze all secondary outcomes by parametric and non-parametric analyses as appropriate, comparing the two randomized groups (access to the MumCare app compared to no access). For the secondary outcome analyses, regression analyses will be performed where relevant, based on directed acyclic graphs (i.e., adjusting for maternal age and BMI in relevant analyses).

## Discussion

There is a lack of research studies evaluating eHealth-assisted CV follow-up of women after pregnancy complications. A personalized follow-up program benefitting from eHealth technology opportunities may have a potential of improving sustainability in healthcare resources and may help empower women to optimize and self-manage their own CV health after pregnancy complications. The MumCare app is, to the best of our knowledge, the first of its kind world-wide and is currently being tested in an RCT in Norway, as described in the present protocol.

Our study provides and tests an innovative eHealth supported follow-up aiming to empower women after pregnancy complications with an epidemiological increased risk of CVD. By promoting individual health optimization postpartum, the need for future specialist CV healthcare will likely be reduced. The study will test whether the use of the MumCare app will promote an improved interaction between the woman herself, her GP and specialist service (obstetricians and midwives), and thereby contributing to narrowing the gap between the health services following pregnancy complications. The long-term goal of our MumCare work is to prevent or postpone maternal CVD. This includes offering guideline-based preventive therapies and strategies at an earlier time point than offered today.

The MumCare study is multidisciplinary, including experts in relevant fields; obstetrics, midwifery, general practice, information technology, cardiology, social sciences, pathology, immunology, epigenetics, neurology, and biomarker research. Patients and user groups have contributed important user perspectives, confirming the need for the study and advising on recruitment and follow-up procedures, as well as testing and improving the app design and functionality. The study enjoys support from three main healthcare provider associations in Norway, providing important user links to Norwegian national health policy makers. This support will facilitate incorporation of MumCare study findings into future revised national guidelines and health services.

One important challenge reported in previous e- and mHealth studies are the low retention rates and adherence to the intervention. Some studies have shown that the use of prompts and proactive reminders may be useful (68–71), which is what we have integrated into the MumCare app. Our co-design approach with users (72) has provided user-activating features (55) that hopefully will result in a high acceptability and longitudinal use in the women randomized to app access in this MumCare RCT.

The primary outcome in this RCT, the rate of CVD preventive follow-up visits at the GP at one year postpartum, was chosen in accordance with the recommendation in the Norwegian guidelines (23, 24, 31, 32). This outcome is relevant in the Norwegian setting, where all inhabitants are provided access to a GP (see study setting above), that also has the life-span perspective of patient-follow-up. Such a study outcome in testing the effects of a postpartum mHealth app may be less relevant for other countries that do not have a public healthcare system, and where the inhabitants are not assigned a GP. Other primary outcomes may be more relevant in such settings, like the ones we have chosen as secondary study outcomes in the MumCare study (e.g., PROMs and objective effects on modifiable CV risk factors, CV biomarkers and CV function). We believe the MumCare study to be of value across different countries, regions and health systems, as mobile phone systems and devices are available in many parts of the world.

A recent Lancet review argued that “Policy makers should consider incorporating reproductive risk factors as part of the assessment of CV risk in clinical guidelines” (73), which is reflected in Norway through national obstetric guidelines (23, 24, 31, 32). We believe the MumCare app represents an effort to implement such guidelines into clinical practice, as the guidelines seem not to be fully followed, nor well-known among GPs, midwives, obstetricians or postpartum women, neither in Norway nor internationally (53, 54). We argue that an eHealth-assisted approach is beneficial, due to the global IT literacy of young women, seeking health information via the internet. We hypothesize that the MumCare app will promote a better collaboration between the woman herself, her GP and specialist service (obstetricians and midwives), thereby reducing the gap in the health services following pregnancy complications.

A follow-up program of maternal CV health after pregnancy complications is most likely to be successful at a population level if it synergizes with other well-functioning public health programs, as suggested in the MumCare study. The MumCare PI’s and collaborators’ previous and ongoing involvement in developing guidelines for their respective Norwegian and international organizations [e.g., The Norwegian College of General Practice, the Norwegian Society of Gynecology and Obstetrics (23, 24, 31, 32) and the Norwegian Society of Cardiology, as well as the International Society for the Study of Hypertension in Pregnancy (26)], is likely to optimize implementation of study results and further guideline and policy development. On that note, we will initiate collaboration through the above mentioned Norwegian associations to improve and update the guidelines from the Norwegian Directorate of Health for primary prevention of CVD after pregnancy complications (56). The wide inclusion of experts, user organization and stakeholders may facilitate incorporation of our MumCare study findings into improved evidence-based guidelines and health services.

### Strengths and limitations of this study

The strengths of the study are summarized above. Our evaluation, from a risk-benefit perspective, is that the MumCare study is well balanced towards the benefit for the individual participant, as well as for the society as a whole. The research project will be conducted in line with national regulations and the international Declaration of Helsinki. The MumCare study and app puts the woman at the center of care.

Increased health competence through app use and optimized maternal health is likely also beneficial for reducing risk of adverse outcomes also in a next pregnancy, representing an added bonus of the MumCare study and app. Another added benefit is the potential effect of an improved lifestyle on the woman’s offspring and partner. The MumCare study provides the woman and the health services with tools to identify modifiable CV risk factors at a young age. This enables early intervention and prophylaxis, reducing the burden on women, families, and the health system, promoting healthier societies, and by this also reducing the need for health personnel.

The study design is aligned with SPIRIT (Standard Protocol Items: recommendations of Intervention Trials) and CONSORT (Consolidated Standards of Reporting Trials).

One study limitation is that some recently immigrated women will be underrepresented in this study due to the need for specific language skills (at present English and Norwegian), limiting the generalizability of the study. The English version of the informed consent was finalized and approved later than the Norwegian consent form, and the inclusion with the English informed consent form started in April 2025, whereas the Norwegian version was used for recruitment since June 2024. Our MumCare app is however considered a first step in providing a more individualized care for women after high-risk pregnancies. Future directions may include more heterogeneous languages, including an Urdu version, as in a previous pregnancy health app tested in Norway (45), reducing potential language barriers for ethnic groups with high rates of gestational diabetes and type 2 diabetes mellitus (e.g., Asian descendants). In addition, we cannot exclude the possibility of a recall or a social desirability bias, leading to recruitment bias. It is well known that people with higher education levels may be more likely to be willing to participate in research studies. We however believe such potential bias to affect both study groups similarly.

A limitation of the current MumCare study is that its primary outcome is best suited for countries with a public healthcare system, where a GP has a leading role in outpatient follow-up. We however envisage that the MumCare app content, and any new follow-up studies, could easily be revised and adapted to other settings than Northern Europe. Another limitation of the primary outcome is that it relies on self-reported information from the participants. Only a small part of the participants will have their information validated through the planned qualitative interviews with their GP, pending patient approvement. The uncertainty in the GP follow-up rates of the two study groups (HDP and GDM), as well as the unknown difference between the two intervention groups (app access or not) is another limitation, challenging the sample size calculations prior to study start.

We expect a successful MumCare app to be disseminated (pending funding opportunities for app maintenance) to the general public through the national health care web site (helsenorge.no), similarly to this website’s linkage to the preeclampsia information brochure for patients (23).

BOX 1 **Language regarding gender.**

**Table:** 

We use in this MumCare protocol and subsequent study publications the terms «woman»/«women» and «women’s health». We acknowledge that not everyone delivering a child identifies with these words or a female gender. The MumCare study is designed to test an mhealth-assisted cardiovascular follow-up for persons that have experienced a pregnancy complication associated with increased risk of premature cardiovascular risk (e.g., preeclampsia, gestational hypertension and gestational diabetes). This means that any pregnant/recently parous persons with these pregnancy complications, also those not identifying with a female gender, are welcome to participate in the MumCare study. The results from the MumCare study may also be relevant to these persons.
