# Supplementary material for: A mobile health-assisted cardiovascular follow-up after pregnancy complications (MumCare): protocol for a randomized controlled trial
Source: Front Glob Womens Health. 2026 Oct 13;7:1889953. doi: 10.3389/fgwh.2026.1889953 (PMC13710215; doi:10.3389/fgwh.2026.1889953)
Supplement: Supplementary file 1 [file Table1.docx]

**Supplemental Table 1.** Examples of expert guidelines for clinical follow-up after adverse pregnancy outcomes for prevention of future cardiovascular disease

| **Publishing Body / Adverse Pregnancy Outcome focus** | **Last Updated** | **Time Point *(from birth)*** | **Recommended Follow-up After Adverse Pregnancy Outcomes** |
| --- | --- | --- | --- |
| **ACOG (Practice Bulletin)^1^**  GH and PE | 2020 | General recommendation | HDP (GH and PE): Preventive strategies and vigilance to be considered by patients and health care providers (healthful weight, exercise, diet, smoking cessation). |
| **AHA^2,3^**  GDM, HDP, placental abruption, preterm birth, SGA | 2024 | Birth-6 weeks | GDM: glucose screening, provide nutritional and exercise counseling.  HDP: early visit, facilitate home BP monitoring.  Assess s-creatinine (usually 1 week postpartum) if history of elevated. |
|  |  | 4-12 weeks | GDM: OGTT |
|  |  | 6-12 months | Assess BMI, BP, Lipids (>3months), glucose, Diet, Sleep, Physical activity, Nicotine exposure. Education on association of APO and long-term CV risk.  HDP: Assess proteinuria (usually around 6-12 months postpartum) if history of elevated. |
|  |  | Annual follow-up | Annual health assessment. |
|  |  | General recommendation | Healthy lifestyle and breastfeeding recommended. CVD risk assessment and follow-up recommended. |
| **Brazilian (FEBRASGO)^4^**  PE | 2019 | Birth-6 weeks | PE: Avoid early discharge (hospital monitoring recommended at least 3 days postpartum).  Reassessment around 7 days postpartum. |
|  |  | General recommendation | Recommends multidisciplinary follow-up, including BP controls, renal function, lipid and glycemic profiles, as well as lifestyle change (physical activity, balanced diet). |
| **Canadian Cardiovascular Society^5^**  HDP, GDM, preterm birth, stillbirth, low birth weight infant, placental abruption | 2021 | 6 weeks-12 months | Screening for elevated blood lipids in the late postpartum period. |
|  |  | General recommendation | Counsel women about increased risk of lifetime CVD after APO, recommendation of healthy lifestyle (healthy body weight, physical activity, smoking cessation, alcohol consumption, stress management, healthy dietary patterns). |
| **CoLab^6^ (Figure S1)**  PE, GH, FGR, GDM | 2016 | Birth-6 weeks | Follow-up of not normalized BP and glucose homeostasis at discharge. |
|  |  | 6-12 weeks | Simple CVD risk assessment at community health follow-up (general practitioner) or specialist according to clinical outcome severity. |
|  |  | General recommendation | Further follow-up and patient education depending on assessed CV risk. |
| **Dutch multidisciplinary guideline^7^**  PE, GH, preterm delivery, SGA, recurrent miscarriage, polycystic ovary syndrome and premature ovarian insufficiency | 2016 | Birth-6 weeks | PE: internist or GP should treat patients using antihypertensive therapy until normal BP without medication achieved. |
|  |  | 6 weeks-49 years | Optimization of lifestyle |
|  |  | At 50 years | PE: full cardiovascular risk profiling |
| **FIGO^8^**  HDP | 2023 | Birth-6 weeks | Monitor BP, BMI, ECG (if available), urine, serum sodium, potassium, creatinine. |
|  |  | 8-12 weeks | Monitor BP, BMI. |
|  |  | 6 months | Cardiovascular and metabolic risk screening: BP, BMI, urinalysis, OGTT, lipid profile. |
|  |  | 12 months | Monitor BP, BMI, lipid profile. Encourage continue breastfeeding, appropriate lifestyle modifications or other interventions if needed. |
|  |  | General recommendation | Follow-up linked to newborn vaccination program: soon after birth, 6 weeks, 6 months and 12 months. Via a multidisciplinary team approach. Inform of risks of recurrence in future pregnancies, lifestyle modifications to help reduce risks, including breastfeeding, dietary and physical activity in every follow-up.  BP assessment every 6-12 months. CV screening at least every 4-6 years**.** |
| **ISSHP^9^**  HDP | 2021 | Birth-1 week | BP monitored at least once a day, 3-7 days postpartum. |
|  |  | 6-12 weeks | Simple CV screening to assess CV risk factors, physical activity and BP. Ensure that BP, urinalysis and any other laboratory abnormalities have normalized. If not refer to further investigations. |
|  |  | 6 months | Clinical assessment, if BP ≥ 120/80 mmHg, lifestyle change. |
|  |  | 12 months | Clinical assessment with additional testing of lipids, blood glucose, HbA1c, CRP and urinary albumin. Lifestyle advice, referral to specialists and drug therapy if needed. |
|  |  | Annual | Medical follow-up first 5-10 years, healthy lifestyle, aim BP <120/80. |
| **NGF**  GDM^10^and HDP^11^ (also referred to the HDP Chapter for CV follow-up in the FGR and Preterm Delivery chapters) | 2020 | 6-12 weeks | CV triage with simple CV risk evaluation (BP, BMI) and recommended healthy lifestyle (physical activity of at least 150 minutes weekly, healthy diet, normal weight, avoid smoking, limit alcohol intake). |
|  |  | 3-6 months | HbA1c after GDM |
|  |  | 12 months | CV triage with extended CV risk evaluation, including lipid assessment and HbA1c. |
|  |  | Following years after 1 years postpartum | Further frequency of follow-up until 50 years of age depends on identified risk factors (e.g. annual to every 5^th^ year follow-up). Follow-up visits include at a minimum simple CV risk evaluation, with BP and lifestyle assessment. |
| **NICE**  HDP^12^ | 2019 (added updates in 2023) | 6-8 weeks | HDP: Medical assessment (GP or specialist)  APE: urinary reagent-strip test 6-8 weeks postpartum. |
|  |  | 3 months | HDP: Those with persistent proteinuria (1+ or more) assess kidney function. |
|  |  | General recommendation | Inform about increased risk of hypertension and CVD.  **Advice:** Normal maternal weight (BMI 18.5-24.9 kg/m^2^) and healthy lifestyle, avoid smoking. |

**Abbreviations**: ACOG: American College of Obstetricians and Gynecologists; AHA: American Heart Association; BMI: body mass index; APO: Adverse pregnancy outcome; BP: blood pressure; CV: Cardiovascular; CVD: Cardiovascular disease; ECG; electrocardiogram; FEBRASGO: Brazilian Federation of Gynecology and Obstetrics Associations; FGR: fetal growth restriction; FIGO: International Federation of Gynecology and Obstetrics; GDM: gestational diabetes mellitus; GH: Gestational hypertension; GP: General practitioner; HbA1c: Hemoglobin A1c; HDP: Hypertensive disorders of pregnancy (includes both preeclampsia and gestational hypertension); ISSHP: The international Society for the Study of Hypertension in Pregnancy; NGF: The Norwegian Society of Gynecology and Obstetrics; NICE: The National Institute for Health and Care Excellence; OGTT: Oral glucose tolerance test; PE: preeclampsia; SGA: Small for gestational age.

**Supplemental Table 1 references:**

1.	Gestational Hypertension and Preeclampsia: ACOG Practice Bulletin, Number 222. *Obstet Gynecol* 2020; **135**(6): e237–e60.

2.	Parikh NI, Gonzalez JM, Anderson CAM, et al. Adverse Pregnancy Outcomes and Cardiovascular Disease Risk: Unique Opportunities for Cardiovascular Disease Prevention in Women: A Scientific Statement From the American Heart Association. *Circulation* 2021; **143**(18): e902–e16.

3.	Lewey J, Beckie TM, Brown HL, et al. Opportunities in the Postpartum Period to Reduce Cardiovascular Disease Risk After Adverse Pregnancy Outcomes: A Scientific Statement From the American Heart Association. *Circulation* 2024; **149**(7): e330–e46.

4.	Peraçoli JC, Borges VTM, Ramos JGL, et al. Pre-eclampsia/Eclampsia. *Rev Bras Ginecol Obstet* 2019; **41**(5): 318–32.

5.	Pearson GJ, Thanassoulis G, Anderson TJ, et al. 2021 Canadian Cardiovascular Society Guidelines for the Management of Dyslipidemia for the Prevention of Cardiovascular Disease in Adults. *Can J Cardiol* 2021; **37**(8): 1129–50.

6.	Staff AC, Redman CW, Williams D, et al. Pregnancy and Long-Term Maternal Cardiovascular Health: Progress Through Harmonization of Research Cohorts and Biobanks. *Hypertension* 2016; **67**(2): 251–60.

7.	Heida KY, Bots ML, de Groot CJ, et al. Cardiovascular risk management after reproductive and pregnancy-related disorders: A Dutch multidisciplinary evidence-based guideline. *European journal of preventive cardiology* 2016; **23**(17): 1863–79.

8.	Poon LC, Nguyen-Hoang L, Smith GN, et al. Hypertensive disorders of pregnancy and long-term cardiovascular health: FIGO Best Practice Advice. *Int J Gynaecol Obstet* 2023; **160 Suppl 1**: 22–34.

9.	Magee LA, Brown MA, Hall DR, et al. The 2021 International Society for the Study of Hypertension in Pregnancy classification, diagnosis & management recommendations for international practice. *Pregnancy Hypertens* 2022; **27**: 148–69.

10.	Staff A SM, Fiskå BF, Friis CM. Gestational diabetes mellitus. Patient information from the Norwegian Society for Gynecology and Obstetrics. 2023. <https://www.legeforeningen.no/contentassets/4afa13a2383d4b0dbce804109efe85b5/ngf-gdm-patient-information-english.pdf>.

11.	Staff A KA, Langseter E, Michelsen TM, Moe K, Strand KM, Værnesbranden M, Øian P. Preeclampsia. Patient Information from the Norwegian Society for Gynecology and Obstetrics. 2020. <https://www.legeforeningen.no/contentassets/b70edd0f6781472db2478b210cbf6e2e/pasientinfo_engelsk_preeklampsi.pdf> (accessed June 22 2022).

12.	Hypertension in pregnancy: diagnosis and management. National Institute for Health and Care Excellence (NICE). 2019. <https://www.nice.org.uk/guidance/ng133> (accessed October 10 2025).
